# Space Radiation Dooms Elon’s Mission to Mars but May Hack the Brain and Fix Depression

**DOI:** 10.7759/cureus.92475

**Published:** 2025-09-16

**Authors:** John R Adler

**Affiliations:** 1 Department of Neurosurgery, Stanford University Medical Center, Stanford, USA; 2 Department of Radiation Oncology, Stanford University School of Medicine, Stanford, USA

**Keywords:** addiction, behavioral disease, depression, functional brain disorders, neuromodulation, psychiatry, radiobiology, radiosurgery

## Abstract

Sorry, Elon, space travel ain’t going to be that simple! There is a growing body of scientific research that indisputably demonstrates the capacity of ionizing radiation to alter the activity of mammalian brain circuits. Unfortunately for the mission to Mars that you are planning, the ubiquitous radiation present throughout interplanetary space is destined to mess with human brain function, and by virtue of such, defeat even the best-engineered rockets and spaceships. Before you make too many more plans for a trip to Mars, it might be a good time to go back to the drawing board and solve this critical human biological reality? Yet still there is reason to celebrate this biological phenomenon; when harnessed correctly, space-like radiation will soon be used here on Earth to treat many common brain disorders, such as depression and addiction, which have plagued us humans through the ages.

## Editorial

Through centuries, the dream of space travel has tantalized humanity. In recent years, Elon Musk elevated this idea to a feverish pitch with his tireless promotion of a manned mission to Mars on one of his SpaceX rockets, possibly even before the end of this decade. To meet this goal, Musk and team have raced to address the daunting engineering challenges needed to build large, powerful rockets, and with each Starship launch, the world anxiously held its collective breath. However, the (willfully) overlooked reality is that the biggest challenge for human Mars travel may not be the engineering. Instead, it is likely to be human physiology, and specifically the brain effects of impossible-to-shield space radiation.

From afar, interplanetary space appears to be merely cold and empty; inhospitable, but not inherently dangerous. However, lurking everywhere in the vacuum of deep space is cosmic radiation, largely in the form of both energetic photons and ionizing particles, which zip around the solar system and galaxy. On Earth, and even to some extent on the International Space Station (ISS), we humans are protected from radiation by the Earth’s electromagnetic field, but get too far away, and radioactive stuff bathes everything. Assuming about 100 days on Mars’ surface, a 2.5-year round trip between Earth and Mars would result in a radiation exposure of 50-100 cGy, depending on exact solar activity [1,2]. While this measure of irradiation may (or not) increase an astronaut’s lifetime risk of cancer, the effect of such radiation doses on brain function cannot be ignored.

For many decades, it was believed that the brain’s “non-dividing” neurons were impervious to all but the largest of radiation doses. In my medical world of stereotactic radiosurgery, where the objective is often ablation, it is widely deemed necessary to give 80-150 Gy doses to get the desired clinical effect; 100X more radiation than the above estimate for a trip to Mars. However, evidence from multiple researchers over the past decade now reveals radiation effects on brain function can be observed in animal models with doses of only 18 cGy, levels of radiation that are well within the range of expected space travel to Mars. Meanwhile, my own (and colleagues') research in large and small mammals like pigs and mice has consistently demonstrated that focal small field non-ablative radiation durably activates selected locally connected brain circuits [3,4]. In other words, radiation isn’t damaging/killing the brain at all, but it is turning on sundry brain circuits, and in doing so, is likely to capriciously affect cognition and behavior. Extrapolate such findings to the entire human brain, and it is uncertain what that might mean. Nevertheless, it is hard to imagine that randomly altering brain function would not scramble an astronaut’s critical executive functioning and higher-level social behaviors. Does this mean space travel is impossible? I don’t know for sure, but the myriad animal data is indisputable, and if I were Mr. Musk, I would abort any near-term manned mission to Mars until I’d come up with a solution!

While my editorial is likely to disappoint many space enthusiasts, including Elon, it comes with a silver lining for us human earthlings. Over the past decade, a host of non-invasive therapies for altering brain function non-invasively have emerged and are increasingly being used to treat refractory psychiatric illnesses. Just maybe the same biology that challenges Musk’s mission to Mars can now be used to precisely alter the faulty brain circuits that cause depression and addiction [5]. Recent animal experiments suggest that radiation doses as low as 5 Gy, a level believed to be thoroughly safe, delivered to minuscule volumes of the brain can induce durable, broad-based changes in neural circuitry. This concept has all the attributes of a near-ideal, non-invasive, simple-to-use treatment for the worst of human afflictions. While the world awaits the exploration of outer space, now is a great time to investigate revolutionary space radiation therapies for us humans’ inner space!
